# Preoperative MRI-based endplate quality: a novel tool for predicting cage subsidence after anterior cervical spine surgery

**DOI:** 10.1186/s13018-024-04716-w

**Published:** 2024-04-16

**Authors:** Yuan Tuo, Kaiyuan Lin, Junsong Yang, Sibo Wang, Haimiti Abudouaini

**Affiliations:** Department of Spine Surgery, Honghui Hospital, Xi’an Jiaotong University, No. 76, Nanguo Road, Beilin District, Xi’an, 710054 Shaanxi China

**Keywords:** Bone mineral density, Cervical spine, Anterior cervical discectomy and fusion, Endplate bone quality, Cage subsidence

## Abstract

**Purpose:**

The objective of this study was to examine the predictive value of a newly developed MRI-based Endplate Bone Quality (EBQ) in relation to the development of cage subsidence following anterior cervical discectomy and fusion (ACDF).

**Methods:**

Patients undergoing ACDF for degenerative cervical diseases between January 2017 and June 2022 were included. Correlation between EBQ scores and segmental height loss was analyzed using Pearson’s correlation. ROC analyses were employed to ascertain the EBQ cut-off values that predict the occurrence of cage subsidence. Multivariate logistic regression analyses were conducted to identify the risk factors associated with postoperative cage subsidence.

**Results:**

23 individuals (14.56%) exhibited the cage subsidence after ACDF. In the nonsubsidence group, the average EBQ and lowest T-score were determined to be 4.13 ± 1.14 and − 0.84 ± 1.38 g/cm^2^ respectively. In contrast, the subsidence group exhibited a mean EBQ and lowest T-score of 5.38 ± 0.47 (*p* < 0.001) and − 1.62 ± 1.34 g/cm^2^ (*p* = 0.014), respectively. There was a significant positive correlation (r = 0.798**) between EBQ and the segmental height loss. The EBQ threshold of 4.70 yielded optimal sensitivity (73.9%) and specificity (93.3%) with AUC of 0.806. Furthermore, the lowest T-score (*p* = 0.045, OR 0.667) and an elevated cervical EBQ score (*p* < 0.001, OR 8.385) were identified as significant risk factors for cage subsidence after ACDF.

**Conclusions:**

The EBQ method presents itself as a promising and efficient tool for surgeons to assess patients at risk of cage subsidence and osteoporosis prior to cervical spine surgery, utilizing readily accessible patient data.

## Introduction

Cage subsidence is currently the most common hardware-related complication of anterior cervical discectomy and fusion (ACDF), and its incidence ranges from 19.3 to 42.5% [1–3]. Postoperative cage subsidence may influence spinal biomechanics and alignment, potentially leading to segmental kyphosis and contributing to adjacent segment disease [1, 2]. Additionally, the reduction in intervertebral space height may result in secondary foraminal stenosis, predisposing to the recurrence of nerve root impingement and radicular symptoms [4, 5]. Several factors have been implicated in the risk of cage subsidence, including patient age, cervical alignment, endplate integrity, bone mineral density (BMD), device type, surgical level and bone graft. Both clinical and biomechanical studies have explored these factors, with BMD recognized as one of the most crucial elements influencing graft subsidence after ACDF [6–10].

The current widely accepted method for assessing bone quality is the evaluation of BMD through the use of dual-energy X-ray absorptiometry (DEXA) [11]. However, recent studies indicated that lumbar fusion surgery patients may experience an overestimation of true T-values during DEXA examination [12–14]. Simultaneously, a dearth of pertinent evidence exists regarding the dependability of utilizing T-values obtained from the hip bone and lumbar spine as a means to estimate the bone mass of the cervical spine. Thus, several alternative techniques have been recently developed to enhance the precision of assessing cervical vertebral bone quality, including the utilization of Hounsfield units (HUs) on CT and MRI-based vertebral bone quality (VBQ) evaluation [12–19]. Although assessing bone quality is a common goal, each measurement has a different focus. For example, the primary objective of DEXA examination is to evaluate the overall bone mass quality, whereas HUs and VBQ are specifically designed to assess the strength of the vertebral bodies. Studies have substantiated that assessments of bone density at specific anatomical sites exhibit greater predictive value for complications compared to conventional measurements encompassing the entire region [20, 21].

In a recent study, Jones et al. [22] designed a pioneering MRI-based evaluation technique for assessing the quality of endplate bone (EBQ) at specific sites. The findings of this study demonstrated that EBQ exhibited a superior predictive capacity compared to alternative approaches in relation to severe cage subsidence subsequent to lumbar spine surgery. However, prior research has predominantly concentrated on the utilization of EBQ with lumbar spine MRI scans in individuals afflicted with lumbar spinal conditions. Based on our current knowledge, there has been no previous investigation conducted on the potential correlation between graft subsidence and the utilization of the EBQ technique in ACDF through the analysis of MRI scans of the cervical spine. Therefore, in this study, we examined the predictive value of this newly developed MRI-based bone evaluation technique in relation to the development of cage subsidence following ACDF and compared with the traditional DEXA examination.

## Methods

### Patient cohort

A retrospective identification was conducted on a cohort of 220 patients who underwent ACDF with the plate cage construct system (Medtronic Sofamor Danek, Inc.) at a single institution within the period of January 2017–February 2022. The inclusion criteria stipulated the availability of at least 1 year of follow-up data, preoperative cervical MRI T1W image and EXA examination. A total of 62 patients were excluded from the study due to incomplete imaging data during the follow-up examination. The remaining 158 patients met the criteria for enrollment, which included being between the ages of 18 and 60, experiencing symptomatic radiculopathy or myelopathy caused by degenerative cervical discs between C3 and C7, and having failed conservative treatment for at least 6 weeks. The exclusion criteria consisted of the following: cervical disc replacement (CDR) or hybrid surgery (CDR with ACDF); ACDF using alternative device types; multilevel surgery; presence of local or systemic infection; pathological vertebral fracture or spinal deformity; allergy to the device material; ankylosing spondylitis; rheumatoid arthritis; or prior cervical spine surgery.

### Surgical procedure

In this study, all patients underwent a Smith-Robinson anterior transcervical approach, which was carried out by a same experienced spinal surgeon. After the initial exposure, the discectomy was performed utilizing the conventional technique, during which the posterior longitudinal ligament and the anterior, posterior, and lateral osteophytes were excised using rongeurs. Following thorough decompression, the all patients were received ACDF utilizing the VENTURE™ anterior cervical plate system (Medtronic Sofamor Danek, Memphis, Tennessee, USA) and a poly-etherether-ketone (PEEK) interbody fusion cage filled with allograft material.

### MRI-based EBQ evaluation

**Fig. 1:**
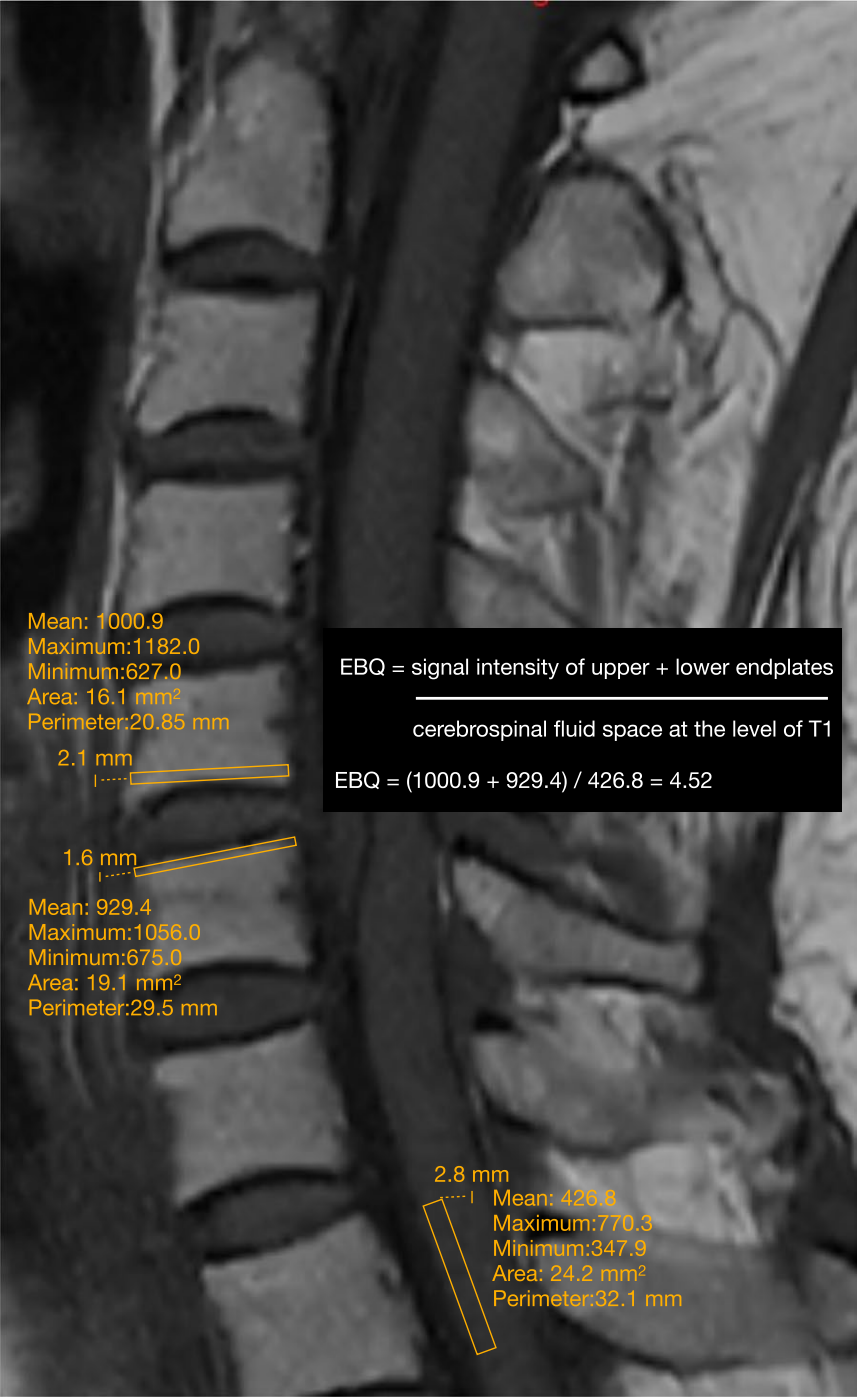
Illustration of the cervical endplate bone quality (EBQ) calculation process: The EBQ assessment is defined as the average value of the signal intensity of the upper and lower endplates at the operated segments, divided by that of the cerebrospinal fluid space at the level of T1

In accordance with the original study conducted by Jones et al. [22], the subchondral bones were identified as the region of interest (ROI) for EBQ measurement. These regions were defined as a 3 mm distance from the upper and lower endplates at the operated level. The average signal intensity of both endplates was then normalized by the signal intensity of the cerebrospinal fluid space at the L3 level. Studies related to the cervical spine, researchers commonly opt to standardize the region of interest (ROI) at the upper T1-level [16]. Therefore, the EBQ assessment was defined as the average value of signal intensity of upper and lower endplates at operated segments divided by that of the cerebrospinal fluid space at the level of T1 on the mid-sagittal MRI T1W slice (Fig. 1). If a Schmorl nodule was detected at the specific level under investigation, the exclusion of the nodule was performed with meticulousness in order to measure the EBQ. The T1-weighted images provide evidence that the signal intensity of fat tissue exceeds that of dense bony tissue. As a result, a higher EBQ score signifies a larger quantity of fat tissue and a smaller quantity of dense bone, thereby establishing an inverse relationship between EBQ and bone density. As a result, a higher EBQ score is indicative of an increased quantity of adipose tissue and a decreased quantity of compact bone, thereby establishing an inverse relationship between EBQ and bone density. Similar to VBQ, a higher EBQ score generally suggests diminished bone strength. The EBQ measurements were performed using the Sectra IDS7 Version 22.1 picture archiving and communication system (PACS) software (Sectra IDS7 Version 22.1, Sectra AB, Link€oping, Sweden).

To address potential measurement errors, a panel of radiologists, consisting of three individuals who were unaware of the study details, was assembled. Two radiologists were assigned the task of collecting data, while the third radiologist was assigned the responsibility of analyzing the data. If any discrepancies exceeding a threshold of 2 were observed between the initial two sets of collected data, the third radiologist was tasked with conducting confirmatory re-measurements.

### Cage subsidence assessment

Segmental height was evaluated through the measurement of the distance between the midpoint of the superior endplate of the upper vertebral body and the midpoint of the inferior endplate of the lower vertebral body, encompassing the fusion site [12]. The measurements were recorded during the initial postoperative radiograph, which took place within one week after the surgical procedure, as well as during the final follow-up. Subsidence was defined as a decrease in segmental height exceeding 2 mm observed during the final follow-up, or the migration of the interbody cage into either endplate, regardless of whether it was accompanied by a reduction in segmental height exceeding 2 mm.

### Bone mineral density

Each patient underwent a dual-energy X-ray absorptiometry at the lumbar spine L2–L4 to assess their bone mineral (L2–L4; in g/cm^2^) density before surgery.

### Statistical analysis

The data was collected and recorded utilizing Microsoft Excel, and subsequently analyzed through the utilization of SPSS Statistics version 21.0 (IBM Corp. Armonk, NY, USA). Descriptive statistics were employed to present the mean and standard deviation for continuous variables, while frequency distribution was utilized for categorical variables. The Student’s *t*-tests were utilized to analyze continuous variables that demonstrated an approximate normal distribution, whereas the Mann–Whitney U tests were employed for variables that exhibited a non-normal distribution. Additionally, the Chi-Square test was applied to compare percentages. First, the data from the two groups were compared and statistically significant variables (*P* < 0.05) and factors that related to the cage subsidence according to the previous literature and our clinical practice. Then, receiver operating characteristic (ROC) curves were generated to determine the optimal cutoff points of cervical EBQ score that were deemed significant in evaluating the probability of cage subsidence. Furthermore, a multivariate logistic regression analysis was conducted to investigate the risk factors associated with cage subsidence following ACDF. The study also obtained 95% confidence intervals and considered *p* < 0.05 (two-sided) as the threshold for statistical significance.

## Results

This study included 158 patients who were followed up for an average duration of 12.15 ± 1.26 months. Of the total patients, 23 individuals (14.56%) experienced cage subsidence, while the remaining 135 patients were categorized as the non-subsidence group. The two groups did not demonstrate statistically significant disparities in terms of age, gender, smoking habits, and body mass index (BMI), as outlined in Table 1.

**Table 1:** Comparison of variables between subsidence and non-subsidence patients

Variable	Non-subsidence	Subsidence	Total	*p*
Patients, n (%)	135	23	158	
Mean age ± SD, years	54.38 ± 9.43	52.73 ± 9.12	54.15 ± 9.36	0.090
Females	62	9	71	
Males	73	14	87	
Smokers	21	3	24	0.749
Nonsmokers	114	20	134	
Mean BMI ± SD, kg/m^2^	23.55 ± 2.77	23.62 ± 2.79	23.56 ± 2.76	0.909
Mean follow-up ± SD, month	12.14 ± 1.03	12.15 ± 1.29	12.15 ± 1.26	0.478
Disc level				
C3/4	12	1	13	
C4/5	22	3	25	
C5/6	84	15	99	
C6/7	17	4	20	
Fusion	134	22	156	
Lowest T-score, g/cm^2^	− 0.84 ± 1.38	− 1.62 ± 1.34	− 0.95 ± 1.40	**0.014**
95% CI	− 1.08 to − 0.61	− 2.20 to − 1.04	− 1.17 to − 0.73	
Mean EBQ score ± SD	4.13 ± 1.14	5.38 ± 0.47	4.31 ± 1.15	**< 0.001**
95% CI	4.01–4.38	4.81–5.02	4.14–4.47	
Mean segmental height loss (mm)	1.05 ± 0.47	2.82 ± 0.31	1.31 ± 0.66	**< 0.001**
95% CI	0.96–1.15	2.61–3.03	1.18–1.44	

Bold value indicates statistically significant differences (*p* < 0.05)

**Fig. 2:**
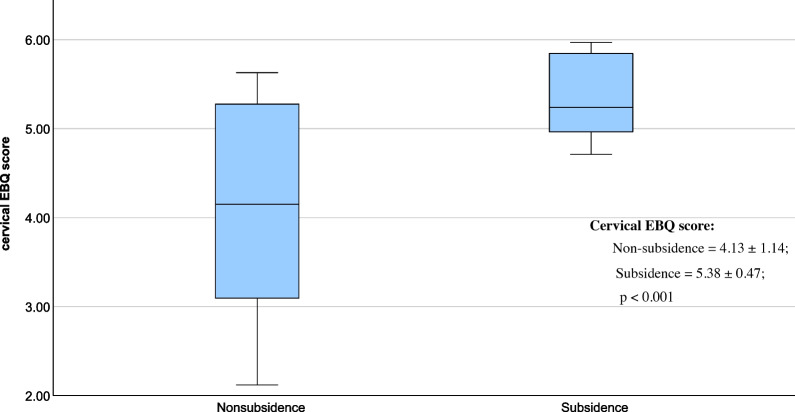
The bar chart illustrates the EBQ score in the subsidence group and non-subsidence group. The average EBQ score was 4.13 ± 0.98 (95% CI 3.94–4.32) in the non-subsidence group and 5.38 ± 0.97 (95% CI 5.18–5.58) in the subsidence group, demonstrating a significant difference in EBQ scores between the two groups (*p* < 0.001)

**Fig. 3:**
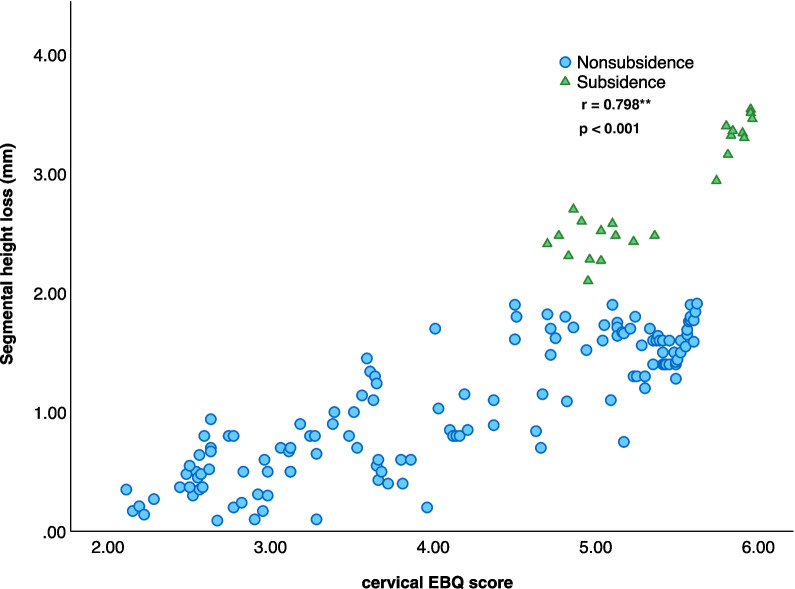
A scatter plot illustrates a strong correlation between the mean EBQ score and postoperative segmental height loss (r = 0.798**, *p* < 0.001)

**Fig. 4:**
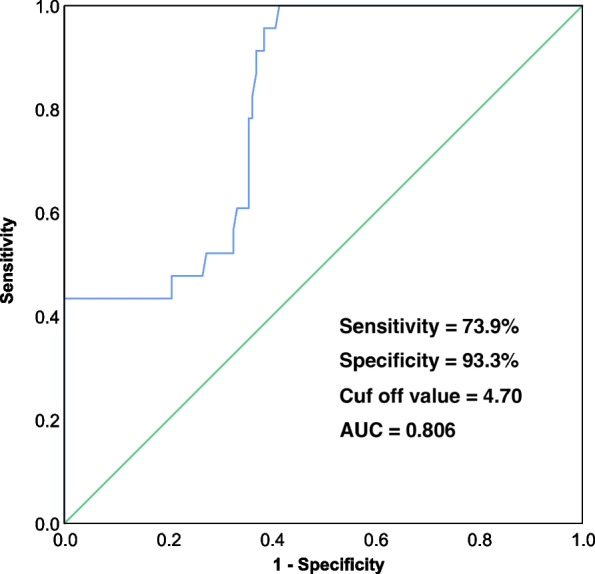
The ROC curve demonstrates sensitivity and specificity for the EBQ score in predicting subsidence after ACDF, with an AUC of 0.806, sensitivity of 73.9%, and specificity of 93.3%

The average EBQ score in the non-subsidence group was 4.13 ± 0.98 (95% CI 3.94–4.32), which exhibited a statistically significant decrease compared to the average EBQ score of the subsidence group (5.38 ± 0.97, 95% CI 5.18–5.58, *p* < 0.001; Table 1, Fig. 2). Additionally, the average loss of segmental height in the non-subsidence group was 1.05 ± 0.47 mm (95% CI 0.96–1.15 mm), while the average loss of segmental height in the subsidence group was 2.82 ± 0.31 mm (95% CI 2.61–3.03 mm; Table 1). A Pearson correlation coefficient analysis was conducted, revealing a statistically significant correlation (r = 0.798**, *p* < 0.001; Fig. 3) between the preoperative EBQ score and postoperative segmental height loss. A ROC curve analysis was conducted to establish the optimal threshold of cervical EBQ score, which was determined to be 4.70 (sensitivity = 73.9%, specificity = 93.3%; AUC = 0.806, 95% CI 0.723–0.890, Fig. 4).

Following a comprehensive multiple logistic regression analysis encompassing variables such as age, sex, smoking status, BMI, preoperative segmental height, lowest T-score, and cervical EBQ score, it was discerned that only a lower T-score (*p* = 0.045, OR 1.499, 95% CI 1.009–2.225) and an elevated cervical EBQ score (*p* < 0.001, OR 0.119, 95% CI 0.038–0.378) were identified as significant risk factors for subsidence (Table 2).

**Table 2:** The results of logistic regression analysis between related factors and subsidence

Covariates	B	P	OR	95% CI	
				Lower bound	Upper bound
Age (years)	0.050	0.116	1.052	0.988	1.120
Sex (females or males)	− 0.145	0.794	0.865	0.291	2.572
Smoking status (yes or no)	− 1.340	0.171	0.262	0.038	1.781
BMI (kg/m^2^)	− 0.094	0.350	0.910	0.748	1.109
Preop segmental height (mm)	− 0.078	0.961	0.925	0.041	20.850
Lowest T-score (g/cm^2^)	0.405	**0.045**	1.499	1.009	2.225
cervical EBQ score	− 2.126	**< 0.001**	0.119	0.038	0.378

Bold value indicates statistically significant differences (*p* < 0.05)

The variability among the sub-axial cervical spine level was assessed by measuring the cervical EBQ score for the patients in the non-subsidence group. The results indicate a gradual decrease in the cervical EBQ score from C3–4 to C6–7. It was found that the cervical EBQ score was gradually decreased from C3–4 to C6–7, the cervical EBQ score of C3–4 was 3.88 ± 1.04 (95% CI 3.22–4.54), C4–5 was 3.92 ± 1.32 (95% CI 3.34–4.51), C5–6 was 4.08 ± 1.11 (95% CI 3.84–4.32) and C6–7 was 4.81 ± 0.91 (95% CI 4.34–5.28; Table 3).

**Table 3:** Mean cervical EBQ score of different subaxial cervical level in patients without subsidence

Level	Mean cervical EBQ score	95% CI	
C3–4 (n = 12)	3.88 ± 1.04	3.22	4.54
C4–5 (n = 22)	3.92 ± 1.32	3.34	4.51
C5–6 (n = 84)	4.08 ± 1.11	3.84	4.32
C6–7 (n = 17)	4.81 ± 0.91	4.34	5.28

## Discussion

In the current literature, a unanimous consensus on the definition of cage subsidence remains elusive. Various criteria have been proposed, with some researchers defining it as a loss of intervertebral height equal to or exceeding 3 mm [7, 23], while others have set the threshold at 2 mm [12, 24–27]. In our study, we adhered to a previously established method for measuring intervertebral height [12], as outlined in the literature, and opted for the widely accepted threshold of 2 mm. The incidence of cage subsidence in this study was 14.56% (23/158), which is consistent with or lower than rates reported in previous studies [28, 29].

As a relatively recent method for assessing bone strength, MRI-based bone quality assessment has garnered attention. Jones et al. [22] conducted a retrospective cohort study to investigate the correlation between EBQ scores and cage subsidence after lumbar interbody fusion. They reported that the average EBQ score was 4.31 ± 1.09 in the non-subsidence group and 5.09 ± 2.20 in the subsidence group. Their EBQ-based model demonstrated superior goodness of fit compared to the VBQ-based model. This finding was corroborated by a recent study by Ai et al. [30], which explored MRI-based VBQ and EBQ scores for assessing bone quality and predicting cage subsidence after TLIF. They found that higher VBQ and EBQ scores were associated with a greater risk of cage subsidence, with EBQ showing greater specificity. In our study, we applied EBQ to assess the quality of cervical endplates. The results revealed that cervical EBQ was an independent predictor of cage subsidence after ACDF. The non-subsidence group exhibited a significantly lower average EBQ score (4.13 ± 0.98) compared to the subsidence group (5.38 ± 0.97). Furthermore, in comparison to the commonly used T-score, the cervical EBQ score showed a stronger correlation with cage subsidence in our study.

Furthermore, our results revealed a significant correlation between the preoperative EBQ score and postoperative segmental height loss, demonstrated by a Pearson correlation coefficient of 0.798 (*p* < 0.001). This correlation strengthens the idea that preoperative assessment of endplate quality can function as a predictive measure for the extent of segmental height loss, contributing to our comprehension of the biomechanical implications of cage subsidence. Importantly, it has been documented that a substantial change in disk height can lead to a significant increase in compressive force between the cage–endplate interface [31, 32]. Consequently, the selection of cage height has been proposed to be determined based on preoperatively measured disk height. Additionally, prior studies have established that cage morphology influences cage subsidence [33, 34]. Specifically, a flat endplate is believed to have better interface contact with an interbody cage surface. This well-matched endplate-cage surface provides more even stress distribution and a larger area for endplate coverage, thereby reducing the incidence of cage subsidence. In contrast, a concave endplate offers a reduced contact area, leading to stress concentration and an increased risk of cage subsidence [35].

While certain authors argue that cage subsidence does not impact clinical outcomes [36, 37], other investigations demonstrate a correlation between graft subsidence and unfavorable clinical outcomes [38–40]. In this study, all patients experienced prompt pain alleviation and neurological improvement following the surgical procedure compared to their preoperative assessments. Notably, no noticeable discrepancies were found in the improvement of Visual Analog Scale (VAS) scores between the subsidence and non-subsidence groups. Furthermore, the absence of a reliable and universally accepted technique for evaluating endplate quality has led to the neglect of endplate degenerative alterations as a potential risk factor for graft subsidence after ACDF. Although this study provides valuable insights into this area, further research is necessary to address existing knowledge gaps. The lack of a widely adopted technique for evaluating endplate quality has contributed to the underestimation of endplate degenerative alterations as a potential risk factor for graft subsidence following ACDF. While the findings reported in this study contribute to filling some data gaps in this field, they are not exhaustive.

This study is subject to several limitations that warrant consideration. Firstly, it is crucial to acknowledge that the research was conducted retrospectively at a single center. To provide a more comprehensive understanding, a prospective randomized controlled study or a multicenter study with a larger sample size would have been preferable. Despite the inclusion of 158 patients, it is noteworthy that the subsidence group comprised only 23 patients. Consequently, the study may have lacked sufficient statistical power to accurately detect genuine disparities due to the limited sample size within the subsidence group. Moreover, while efforts were made to minimize measurement errors by establishing an independent panel of three study-blinded radiologists for EBQ measurements, it is essential to recognize that radiographic imaging errors could have influenced our results. Another limitation lies in the absence of consistent vertebral bone quality data, such as Hounsfield Unit (HU) values measured on CT and MRI-based Vertebral Bone Quality (VBQ). These methods were not included in the logistic regression analysis for several reasons. Firstly, it was not routine to order preoperative CT scans before a single-level ACDF in our hospital. Additionally, conducting a multifactorial analysis incorporating each of these bone assessment methods and risk factors in a single retrospective study presented logistical challenges. The primary focus of this study was to evaluate the correlation between cage subsidence and EBQ values. Future studies should delve into this relationship and undertake a more comprehensive multifactorial analysis.

An additional limitation of this study lies in the selection of clinical outcome measures. Specifically, we only included the Visual Analog Scale (VAS) score, while omitting the Japanese Orthopaedic Association (JOA) score and Neck Disability Index (NDI). Consequently, the assessment of postoperative neurological functions and the evaluation of activities of daily living and movement disorders were not undertaken. This limitation restricts a comprehensive understanding of the broader impact of surgical intervention. Furthermore, the study did not provide effective preoperative and intra-operative methods aimed at reducing the subsidence rate. Future investigations should prioritize the exploration of practical techniques to mitigate implant-related complications and minimize the need for revision surgery following Anterior Cervical Discectomy and Fusion (ACDF), particularly in patients with a high EBQ score.

## Conclusion

In this study, the EBQ method was initially utilized to evaluate the BMD in patients undergoing cervical spine surgery, using preoperative cervical spine MRI. The presence of high EBQ was identified as an independent predictor for cage subsidence following ACDF, demonstrating superior predictive capability compared to the conventional bone assessment method. Hence, the EBQ method presents itself as a promising and efficient tool for surgeons to assess patients at risk of cage subsidence and osteoporosis prior to cervical spine surgery, utilizing readily accessible patient data.

## Data Availability

The datasets utilized and/or examined in the present study can be obtained from the corresponding author upon a reasonable request.
